# Methyl 1-allyl-4-hy­droxy-2,2-dioxo-1*H*-2λ^6^,1-benzo­thia­zine-3-carboxyl­ate

**DOI:** 10.1107/S1600536813028572

**Published:** 2013-10-23

**Authors:** Svitlana V. Shishkina, Igor V. Ukrainets, Lidiya A. Petrushova

**Affiliations:** a STC "Institute for Single Crystals", National Academy of Sciences of Ukraine, 60 Lenina ave., Kharkiv 61001, Ukraine; b National University of Pharmacy, 4 Blyukhera St, Kharkiv 61168, Ukraine

## Abstract

There are two independent mol­ecules in the asymmetric unit of the title compound, C_13_H_13_NO_5_S, in both of which the ester substituent is nearly coplanar [C—C—C—O torsion angles = 2.7 (7) and −0.8 (7)°] with the planar fragment of the bicycle due to the formation of a strong O—H⋯O intra­molecular hydrogen bond. The vinyl group at the ring N atom is approximately orthogonal to the heterocyclic mean plane [C—N—C—C torsion angles = 103.1 (6) and 98.2 (5)°]. The refinement was performed on a two-component, non-merohedrally twinned crystal [population ratio = 0.483 (3):0.517 (3).

## Related literature

For general properties of oxicams, see: Kleemann *et al.* (2008 ▶). For H⋯O contacts, see: Zefirov (1997 ▶) and for C—N bond lengths, see: Bürgi & Dunitz (1994 ▶).
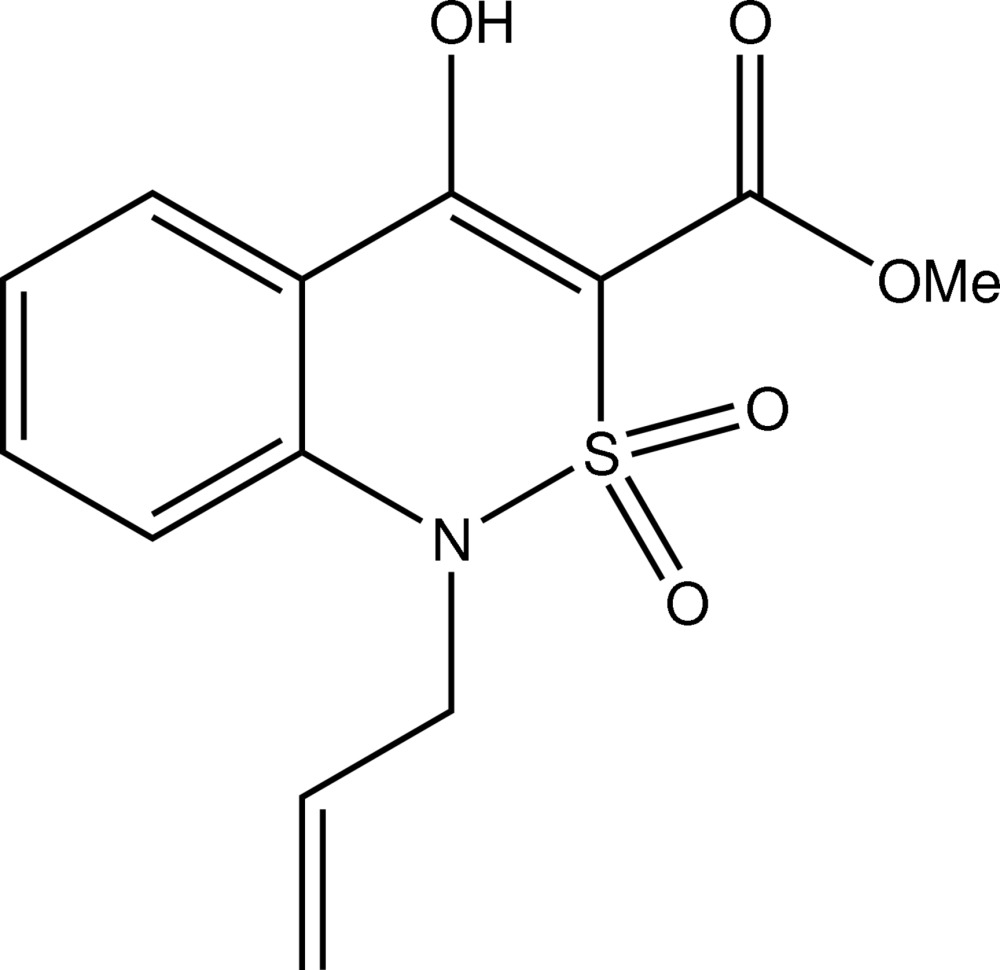


## Experimental

### 

#### Crystal data

C_13_H_13_NO_5_S
*M* *_r_* = 295.30
Monoclinic, 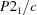

*a* = 17.8654 (12) Å
*b* = 6.9444 (5) Å
*c* = 21.1462 (16) Å
β = 90.122 (7)°
*V* = 2623.5 (3) Å^3^
*Z* = 8
Mo *K*α radiation
μ = 0.27 mm^−1^
*T* = 293 K
0.30 × 0.10 × 0.10 mm

#### Data collection

Agilent Xcalibur3 diffractometer
Absorption correction: multi-scan (*CrysAlis RED*; Agilent, 2011 ▶) *T*_min_ = 0.925, *T*_max_ = 0.974
4647 measured reflections
4647 independent reflections
3728 reflections with *I* > 2σ(*I*)

#### Refinement

*R*[*F* ^2^ > 2σ(*F* ^2^)] = 0.069
*wR*(*F* ^2^) = 0.218
*S* = 1.15
4647 reflections
366 parameters
H-atom parameters constrained
Δρ_max_ = 0.41 e Å^−3^
Δρ_min_ = −0.40 e Å^−3^

### 

Data collection: *CrysAlis CCD* (Agilent, 2011 ▶); cell refinement: *CrysAlis CCD*; data reduction: *CrysAlis RED* (Agilent, 2011 ▶); program(s) used to solve structure: *SHELXTL* (Sheldrick, 2008 ▶); program(s) used to refine structure: *SHELXTL*; molecular graphics: *XP* in *SHELXTL*; software used to prepare material for publication: *SHELXTL*.

## Supplementary Material

Crystal structure: contains datablock(s) I, global. DOI: 10.1107/S1600536813028572/bg2518sup1.cif

Structure factors: contains datablock(s) I. DOI: 10.1107/S1600536813028572/bg2518Isup2.hkl

Supplementary material file. DOI: 10.1107/S1600536813028572/bg2518Isup3.cml

Additional supplementary materials:  crystallographic information; 3D view; checkCIF report

## Figures and Tables

**Table 1:** Hydrogen-bond geometry (Å, °)

*D*—H⋯*A*	*D*—H	H⋯*A*	*D*⋯*A*	*D*—H⋯*A*
O1*A*—H1*OA*⋯O2*A*	0.82	1.84	2.553 (8)	144
O1*B*—H1*OB*⋯O2*B*	0.82	1.87	2.588 (8)	146

**Fig. 1.**
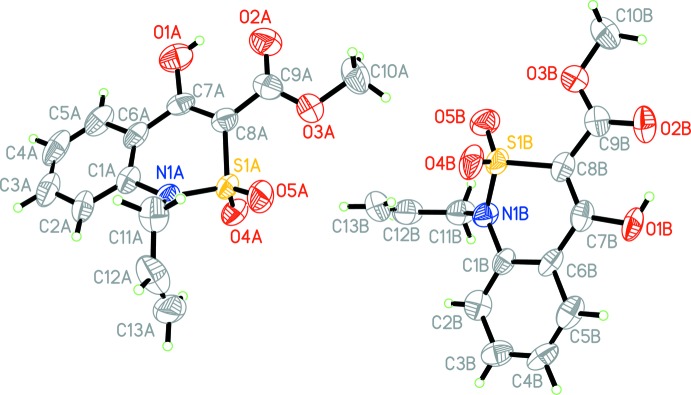
View of the title compound with atomic membering. All atoms are shown with displacement ellipsoids drawn at the 50% probability level.

## supplementary crystallographic information

### 1. Comment

Oxicams are in integral part of the range of modern non-steroidal anti-inflammatory drugs (Kleemann *et al.*, 2008). We have carried out the synthesis and studied the peculiarities of the spatial structure of methyl 4-hydroxy-1-methyl-2,2-dioxo-1*H*-2λ^6^,1-benzothiazine-3-carboxylate (I) being of interest as the initial product for obtaining 4-hydroxy-2,2-dioxo-1*H*- 2λ^6^,1-benzothiazine-3-carboxamides. By now these compounds remain absolutely unstudied though they are isomers of oxicams and differ from them only by the reverse mutual arrangement of nitrogen and sulfur atoms in the thiazine cycle. Two molecules (IA and IB) are observed in asymmetric part of crystal unit cell. The dihydrothiazine heterocycle adopts an intermediate between twist-boat and sofa conformation (the puckering parameters [1] are: S=0.64, Θ=55.8°, Ψ=22.9° for IA and S=0.61, Θ=50.2°, Ψ=21.1° for IB). Deviations of the S1 and C8 atoms from the mean plane of the remaining atoms of this ring are 0.91 Å and 0.29 Å, respectivey, in IA and -0.84 Å and -0.23 Å, in IB. The formation of the strong O1—H···O2 hydrogen bond (Table 1) results in coplanarity of the ester substituent to the C7—C8 endocyclic double bond (the C7—C8—C9—O2 torsion angle is 2.7 (7)° in IA and -0.8 (7)° in IB). The vinyl fragment is orthogonal to the hetetocyclic plane and is coplanar to the N1—C11 bond (the C1—N1—C11—C12 and N1—C11—C12—C13 torsion angles are 103.1 (6)° IA 98.2 (5)° IB and 9.3 (9)° IA 8.5 (9)%A IB, respectively). The repulsion between allyl substituent and atoms of the bicyclic fragment [the H2···C11 distance is 2.76Å IA 2.70Å IB, H11*a*···C2 2.76Å IA 2.71° IB, H11*b*···O5 2.41Å IA 2.38Å IB as compared to the van der Waals radii sum 2.87 Å for H···C contact and 2.46 Å for H···O (Zefirov, 1997)] results in elongation of the C1—N1 bond up to 1.416 (6) Å in IA and 1.414 (6) Å in IB while its mean value is 1.371 Å (Bürgi & Dunitz, 1994).

### 2. Experimental

Triethylamine (1.54 ml, 11 mmol) was added to the solution of methyl *N*-allylanthranilate (1.91 g, 10 mmol) in CH_2_Cl_2_ (20 ml). Chlorosulfonyl acetic acid ethyl ester (2.05 g, 11 mol) was then added dropwise with cooling and stirring and left at the room temperature for 5 h. The reaction mixture was diluted with a cold water and vigorously stirred. The organic layer was separated, dried over CaCl_2_, and the solvent was distilled off (finally under reduced pressure). The residue was treated with the solution of sodium methylate (prepared from metallic sodium (0.69 g, 30 mmol) and absolute MeOH (15 ml)), taken fo reflux, and then left for 10–12 h at the room temperature. The reaction mixture was diluted with cold water and acidified with 1 N HCl to pH 3. The precipitate was filtered, washed with water, and dried. Yield 2.39 g (81%). *M*.p. 114–116%A C (MeOH).

### 3. Refinement

The refinement was performed on a two-component, non-merohedral twinned crystal (Refined populations: 0.483 (3),0.517 (3) ). All hydrogen atoms were located from electron density difference maps and were refined in the riding motion approximation with U_iso_ constrained to be 1.5 times U_eq_ of the carrier atom for the methyl and hydroxyl groups and 1.2 times U_eq_ of the carrier atom for the other atoms.

### Crystal data

**Table:**

C_13_H_13_NO_5_S	*F*(000) = 1232
*M**_r_* = 295.30	*D*_x_ = 1.495 Mg m^−^^3^
Monoclinic, *P*2_1_/*c*	Mo *K*α radiation, λ = 0.71073 Å
*a* = 17.8654 (12) Å	Cell parameters from 4369 reflections
*b* = 6.9444 (5) Å	θ = 2.9–32.0°
*c* = 21.1462 (16) Å	µ = 0.27 mm^−^^1^
β = 90.122 (7)°	*T* = 293 K
*V* = 2623.5 (3) Å^3^	Stick, colourless
*Z* = 8	0.30 × 0.10 × 0.10 mm

### Data collection

**Table:**

Agilent Xcalibur3 diffractometer	4647 independent reflections
Radiation source: Enhance (Mo) X-ray Source	3728 reflections with *I* > 2σ(*I*)
Graphite monochromator	*R*_int_ = 0.000
Detector resolution: 16.1827 pixels mm^-1^	θ_max_ = 25.0°, θ_min_ = 3.0°
ω scans	*h* = −19→21
Absorption correction: multi-scan (*CrysAlis RED*; Agilent, 2011)	*k* = −8→8
*T*_min_ = 0.925, *T*_max_ = 0.974	*l* = −25→25
4647 measured reflections	

### Refinement

**Table:**

Refinement on *F*^2^	Primary atom site location: structure-invariant direct methods
Least-squares matrix: full	Secondary atom site location: difference Fourier map
*R*[*F*^2^ > 2σ(*F*^2^)] = 0.069	Hydrogen site location: difference Fourier map
*wR*(*F*^2^) = 0.218	H-atom parameters constrained
*S* = 1.15	*w* = 1/[σ^2^(*F*_o_^2^) + (0.1383*P*)^2^ + 0.312*P*] where *P* = (*F*_o_^2^ + 2*F*_c_^2^)/3
4647 reflections	(Δ/σ)_max_ = 0.001
366 parameters	Δρ_max_ = 0.41 e Å^−^^3^
0 restraints	Δρ_min_ = −0.40 e Å^−^^3^

### Special details

**Table:**

Geometry. All e.s.d.'s (except the e.s.d. in the dihedral angle between two l.s. planes) are estimated using the full covariance matrix. The cell e.s.d.'s are taken into account individually in the estimation of e.s.d.'s in distances, angles and torsion angles; correlations between e.s.d.'s in cell parameters are only used when they are defined by crystal symmetry. An approximate (isotropic) treatment of cell e.s.d.'s is used for estimating e.s.d.'s involving l.s. planes.
Refinement. Refinement of *F*^2^ against ALL reflections. The weighted *R*-factor *wR* and goodness of fit *S* are based on *F*^2^, conventional *R*-factors *R* are based on *F*, with *F* set to zero for negative *F*^2^. The threshold expression of *F*^2^ > σ(*F*^2^) is used only for calculating *R*-factors(gt) *etc*. and is not relevant to the choice of reflections for refinement. *R*-factors based on *F*^2^ are statistically about twice as large as those based on *F*, and *R*- factors based on ALL data will be even larger.

### Fractional atomic coordinates and isotropic or equivalent isotropic displacement parameters (Å^2^)

**Table:**

	*x*	*y*	*z*	*U*_iso_*/*U*_eq_	
S1A	0.14036 (9)	0.3858 (2)	0.17531 (7)	0.0470 (4)	
N1A	0.1098 (3)	0.2473 (7)	0.1181 (3)	0.0444 (12)	
O1A	−0.0661 (3)	0.3675 (8)	0.2396 (3)	0.0712 (15)	
H1OA	−0.0543	0.3505	0.2766	0.107*	
O2A	0.0229 (3)	0.3701 (8)	0.3341 (2)	0.0733 (14)	
O3A	0.1452 (3)	0.3964 (7)	0.3154 (2)	0.0612 (12)	
O4A	0.1432 (3)	0.5799 (7)	0.1529 (2)	0.0626 (12)	
O5A	0.2044 (3)	0.2977 (9)	0.1996 (2)	0.0737 (15)	
C1A	0.0368 (3)	0.2867 (8)	0.0946 (3)	0.0446 (14)	
C2A	0.0214 (4)	0.2520 (9)	0.0319 (3)	0.0545 (17)	
H2A	0.0580	0.2029	0.0051	0.065*	
C3A	−0.0493 (4)	0.2915 (11)	0.0099 (4)	0.068 (2)	
H3A	−0.0600	0.2716	−0.0326	0.081*	
C4A	−0.1044 (4)	0.3594 (10)	0.0488 (5)	0.072 (2)	
H4A	−0.1515	0.3874	0.0323	0.087*	
C5A	−0.0903 (4)	0.3864 (9)	0.1120 (4)	0.0579 (18)	
H5A	−0.1282	0.4285	0.1387	0.070*	
C6A	−0.0196 (4)	0.3509 (8)	0.1358 (3)	0.0455 (14)	
C7A	−0.0051 (4)	0.3652 (8)	0.2037 (3)	0.0445 (15)	
C8A	0.0646 (4)	0.3710 (8)	0.2297 (3)	0.0464 (15)	
C9A	0.0758 (5)	0.3764 (9)	0.2972 (3)	0.0534 (17)	
C10A	0.1605 (5)	0.3986 (11)	0.3825 (3)	0.072 (2)	
H10A	0.2111	0.4399	0.3896	0.108*	
H10B	0.1538	0.2715	0.3994	0.108*	
H10C	0.1267	0.4859	0.4030	0.108*	
C11A	0.1484 (5)	0.0682 (10)	0.1012 (3)	0.0633 (18)	
H11A	0.1116	−0.0235	0.0861	0.076*	
H11B	0.1709	0.0147	0.1392	0.076*	
C12A	0.2079 (5)	0.0896 (18)	0.0522 (4)	0.089 (3)	
H12A	0.2286	−0.0229	0.0360	0.106*	
C13A	0.2336 (5)	0.255 (2)	0.0298 (4)	0.107 (4)	
H13A	0.2144	0.3708	0.0446	0.128*	
H13B	0.2709	0.2549	−0.0009	0.128*	
S1B	0.36187 (9)	0.7281 (2)	0.43097 (7)	0.0483 (4)	
N1B	0.3887 (3)	0.8759 (7)	0.3742 (2)	0.0462 (13)	
O1B	0.5691 (2)	0.7495 (7)	0.4896 (2)	0.0588 (13)	
H1OB	0.5583	0.7425	0.5272	0.088*	
O2B	0.4815 (3)	0.7162 (7)	0.5863 (2)	0.0655 (13)	
O3B	0.3585 (3)	0.7152 (7)	0.5704 (2)	0.0617 (12)	
O4B	0.3600 (3)	0.5376 (6)	0.4066 (2)	0.0643 (13)	
O5B	0.2942 (3)	0.8056 (8)	0.4570 (2)	0.0695 (14)	
C1B	0.4624 (3)	0.8560 (8)	0.3489 (3)	0.0409 (14)	
C2B	0.4758 (4)	0.9081 (9)	0.2867 (3)	0.0539 (17)	
H2B	0.4370	0.9524	0.2613	0.065*	
C3B	0.5496 (4)	0.8934 (9)	0.2621 (4)	0.0562 (17)	
H3B	0.5592	0.9245	0.2201	0.067*	
C4B	0.6055 (4)	0.8337 (9)	0.3004 (4)	0.0557 (17)	
H4B	0.6539	0.8252	0.2844	0.067*	
C5B	0.5925 (4)	0.7856 (9)	0.3619 (4)	0.0539 (17)	
H5B	0.6322	0.7447	0.3870	0.065*	
C6B	0.5194 (3)	0.7966 (7)	0.3885 (3)	0.0417 (13)	
C7B	0.5071 (4)	0.7615 (8)	0.4556 (3)	0.0469 (15)	
C8B	0.4367 (4)	0.7462 (8)	0.4823 (3)	0.0465 (15)	
C9B	0.4276 (4)	0.7230 (8)	0.5511 (3)	0.0496 (16)	
C10B	0.3435 (5)	0.7079 (11)	0.6370 (3)	0.068 (2)	
H10D	0.3152	0.8194	0.6491	0.102*	
H10E	0.3899	0.7054	0.6599	0.102*	
H10F	0.3153	0.5938	0.6464	0.102*	
C11B	0.3386 (4)	1.0290 (10)	0.3539 (3)	0.0561 (17)	
H11C	0.3688	1.1344	0.3381	0.067*	
H11D	0.3120	1.0760	0.3908	0.067*	
C12B	0.2831 (4)	0.9795 (16)	0.3054 (4)	0.077 (3)	
H12B	0.2564	1.0801	0.2872	0.093*	
C13B	0.2678 (5)	0.799 (2)	0.2850 (4)	0.096 (3)	
H13C	0.2934	0.6944	0.3021	0.116*	
H13D	0.2317	0.7794	0.2539	0.116*	

### Atomic displacement parameters (Å^2^)

**Table:**

	*U*^11^	*U*^22^	*U*^33^	*U*^12^	*U*^13^	*U*^23^
S1A	0.0278 (7)	0.0627 (9)	0.0505 (7)	−0.0049 (7)	−0.0033 (8)	−0.0054 (7)
N1A	0.030 (2)	0.048 (3)	0.055 (3)	0.002 (2)	−0.001 (2)	−0.005 (2)
O1A	0.049 (3)	0.080 (4)	0.084 (3)	0.004 (2)	0.012 (3)	0.003 (3)
O2A	0.077 (4)	0.084 (4)	0.059 (3)	−0.007 (3)	0.018 (3)	−0.011 (2)
O3A	0.064 (3)	0.072 (3)	0.048 (2)	−0.007 (3)	−0.001 (3)	−0.005 (2)
O4A	0.057 (3)	0.062 (3)	0.068 (3)	−0.026 (2)	0.004 (3)	−0.004 (2)
O5A	0.035 (3)	0.123 (5)	0.063 (3)	0.007 (3)	−0.012 (2)	−0.011 (3)
C1A	0.037 (3)	0.038 (3)	0.059 (4)	−0.002 (3)	−0.009 (3)	0.003 (3)
C2A	0.048 (4)	0.052 (3)	0.063 (4)	−0.010 (3)	−0.021 (3)	0.001 (3)
C3A	0.064 (5)	0.059 (4)	0.080 (5)	−0.026 (4)	−0.029 (5)	0.013 (4)
C4A	0.046 (4)	0.064 (4)	0.107 (7)	−0.015 (4)	−0.036 (5)	0.029 (4)
C5A	0.034 (3)	0.045 (3)	0.094 (5)	0.005 (3)	−0.009 (4)	0.010 (3)
C6A	0.039 (3)	0.031 (3)	0.066 (4)	−0.008 (3)	−0.005 (3)	0.007 (3)
C7A	0.033 (3)	0.040 (3)	0.060 (4)	0.002 (2)	0.013 (3)	0.000 (3)
C8A	0.042 (4)	0.042 (3)	0.055 (3)	−0.003 (3)	0.009 (3)	−0.006 (3)
C9A	0.072 (5)	0.040 (3)	0.048 (3)	−0.003 (3)	0.011 (4)	−0.002 (3)
C10A	0.107 (7)	0.056 (4)	0.053 (4)	−0.004 (4)	−0.015 (4)	−0.009 (3)
C11A	0.065 (5)	0.054 (4)	0.071 (4)	0.011 (4)	−0.009 (4)	−0.011 (3)
C12A	0.056 (5)	0.149 (9)	0.061 (5)	0.057 (6)	−0.011 (4)	−0.035 (5)
C13A	0.043 (5)	0.218 (13)	0.058 (5)	−0.023 (7)	−0.007 (4)	0.019 (7)
S1B	0.0336 (8)	0.0578 (9)	0.0535 (8)	−0.0016 (8)	−0.0105 (8)	0.0126 (7)
N1B	0.036 (3)	0.049 (3)	0.053 (3)	0.006 (2)	0.000 (2)	0.012 (2)
O1B	0.032 (2)	0.074 (3)	0.071 (3)	0.003 (2)	−0.022 (2)	0.003 (3)
O2B	0.060 (3)	0.076 (3)	0.061 (3)	0.012 (3)	−0.027 (3)	−0.005 (2)
O3B	0.057 (3)	0.079 (3)	0.049 (2)	−0.010 (3)	0.001 (3)	0.000 (2)
O4B	0.064 (3)	0.051 (2)	0.078 (3)	−0.027 (2)	−0.027 (3)	0.013 (2)
O5B	0.040 (3)	0.099 (4)	0.070 (3)	0.006 (2)	0.001 (2)	0.023 (3)
C1B	0.032 (3)	0.035 (3)	0.056 (3)	0.003 (2)	−0.007 (3)	0.004 (3)
C2B	0.057 (4)	0.041 (3)	0.063 (4)	0.007 (3)	−0.006 (3)	0.005 (3)
C3B	0.055 (4)	0.042 (3)	0.071 (4)	−0.006 (3)	0.014 (4)	0.002 (3)
C4B	0.031 (3)	0.048 (4)	0.088 (5)	−0.001 (3)	0.012 (3)	−0.009 (4)
C5B	0.038 (4)	0.037 (3)	0.086 (5)	0.010 (3)	−0.009 (4)	−0.007 (3)
C6B	0.027 (3)	0.030 (3)	0.069 (4)	−0.002 (2)	−0.003 (3)	−0.005 (3)
C7B	0.043 (4)	0.036 (3)	0.062 (4)	0.005 (3)	−0.014 (3)	−0.004 (3)
C8B	0.040 (4)	0.037 (3)	0.063 (4)	−0.001 (2)	−0.011 (3)	0.002 (3)
C9B	0.057 (4)	0.039 (3)	0.053 (3)	0.004 (3)	−0.008 (3)	−0.005 (3)
C10B	0.091 (6)	0.068 (4)	0.046 (3)	0.002 (4)	−0.004 (4)	−0.004 (3)
C11B	0.044 (4)	0.060 (4)	0.065 (4)	0.017 (3)	0.006 (3)	0.014 (3)
C12B	0.039 (4)	0.130 (8)	0.063 (4)	0.017 (4)	−0.002 (3)	0.041 (5)
C13B	0.074 (6)	0.166 (11)	0.049 (4)	−0.024 (6)	−0.013 (4)	0.008 (5)

### Geometric parameters (Å, º)

**Table:**

S1A—O5A	1.394 (5)	S1B—O4B	1.420 (5)
S1A—O4A	1.430 (5)	S1B—O5B	1.435 (6)
S1A—N1A	1.638 (5)	S1B—N1B	1.651 (5)
S1A—C8A	1.782 (7)	S1B—C8B	1.724 (6)
N1A—C1A	1.421 (7)	N1B—C1B	1.429 (8)
N1A—C11A	1.467 (8)	N1B—C11B	1.454 (8)
O1A—C7A	1.330 (8)	O1B—C7B	1.321 (7)
O1A—H1OA	0.8200	O1B—H1OB	0.8200
O2A—C9A	1.226 (9)	O2B—C9B	1.217 (8)
O3A—C9A	1.305 (9)	O3B—C9B	1.303 (8)
O3A—C10A	1.445 (8)	O3B—C10B	1.434 (8)
C1A—C2A	1.374 (9)	C1B—C6B	1.382 (8)
C1A—C6A	1.406 (9)	C1B—C2B	1.385 (9)
C2A—C3A	1.373 (10)	C2B—C3B	1.422 (10)
C2A—H2A	0.9300	C2B—H2B	0.9300
C3A—C4A	1.367 (12)	C3B—C4B	1.349 (10)
C3A—H3A	0.9300	C3B—H3B	0.9300
C4A—C5A	1.373 (12)	C4B—C5B	1.364 (10)
C4A—H4A	0.9300	C4B—H4B	0.9300
C5A—C6A	1.381 (9)	C5B—C6B	1.426 (9)
C5A—H5A	0.9300	C5B—H5B	0.9300
C6A—C7A	1.464 (9)	C6B—C7B	1.456 (9)
C7A—C8A	1.359 (9)	C7B—C8B	1.385 (10)
C8A—C9A	1.442 (9)	C8B—C9B	1.472 (10)
C10A—H10A	0.9600	C10B—H10D	0.9600
C10A—H10B	0.9600	C10B—H10E	0.9600
C10A—H10C	0.9600	C10B—H10F	0.9600
C11A—C12A	1.493 (13)	C11B—C12B	1.465 (11)
C11A—H11A	0.9700	C11B—H11C	0.9700
C11A—H11B	0.9700	C11B—H11D	0.9700
C12A—C13A	1.325 (16)	C12B—C13B	1.354 (15)
C12A—H12A	0.9300	C12B—H12B	0.9300
C13A—H13A	0.9300	C13B—H13C	0.9300
C13A—H13B	0.9300	C13B—H13D	0.9300
			
O5A—S1A—O4A	120.4 (3)	O4B—S1B—O5B	118.0 (3)
O5A—S1A—N1A	106.6 (3)	O4B—S1B—N1B	108.8 (3)
O4A—S1A—N1A	108.7 (3)	O5B—S1B—N1B	106.9 (3)
O5A—S1A—C8A	111.2 (3)	O4B—S1B—C8B	108.3 (3)
O4A—S1A—C8A	107.3 (3)	O5B—S1B—C8B	112.6 (3)
N1A—S1A—C8A	101.0 (3)	N1B—S1B—C8B	100.8 (3)
C1A—N1A—C11A	120.7 (5)	C1B—N1B—C11B	121.8 (5)
C1A—N1A—S1A	116.7 (4)	C1B—N1B—S1B	118.8 (4)
C11A—N1A—S1A	121.4 (4)	C11B—N1B—S1B	119.3 (4)
C7A—O1A—H1OA	109.5	C7B—O1B—H1OB	109.5
C9A—O3A—C10A	117.9 (6)	C9B—O3B—C10B	119.3 (6)
C2A—C1A—C6A	120.7 (6)	C6B—C1B—C2B	121.6 (6)
C2A—C1A—N1A	119.0 (6)	C6B—C1B—N1B	118.7 (5)
C6A—C1A—N1A	120.2 (6)	C2B—C1B—N1B	119.5 (5)
C3A—C2A—C1A	118.3 (8)	C1B—C2B—C3B	119.5 (6)
C3A—C2A—H2A	120.9	C1B—C2B—H2B	120.3
C1A—C2A—H2A	120.9	C3B—C2B—H2B	120.3
C4A—C3A—C2A	121.9 (8)	C4B—C3B—C2B	119.3 (7)
C4A—C3A—H3A	119.0	C4B—C3B—H3B	120.4
C2A—C3A—H3A	119.0	C2B—C3B—H3B	120.4
C3A—C4A—C5A	120.1 (7)	C3B—C4B—C5B	121.3 (6)
C3A—C4A—H4A	120.0	C3B—C4B—H4B	119.4
C5A—C4A—H4A	120.0	C5B—C4B—H4B	119.4
C4A—C5A—C6A	119.7 (8)	C4B—C5B—C6B	121.5 (6)
C4A—C5A—H5A	120.1	C4B—C5B—H5B	119.2
C6A—C5A—H5A	120.1	C6B—C5B—H5B	119.2
C5A—C6A—C1A	119.2 (6)	C1B—C6B—C5B	116.8 (6)
C5A—C6A—C7A	120.4 (7)	C1B—C6B—C7B	121.9 (6)
C1A—C6A—C7A	120.2 (6)	C5B—C6B—C7B	121.1 (6)
O1A—C7A—C8A	121.4 (6)	O1B—C7B—C8B	122.3 (6)
O1A—C7A—C6A	114.6 (6)	O1B—C7B—C6B	114.4 (6)
C8A—C7A—C6A	124.0 (6)	C8B—C7B—C6B	123.3 (6)
C7A—C8A—C9A	121.7 (7)	C7B—C8B—C9B	120.8 (6)
C7A—C8A—S1A	115.9 (5)	C7B—C8B—S1B	116.9 (5)
C9A—C8A—S1A	122.3 (6)	C9B—C8B—S1B	121.8 (5)
O2A—C9A—O3A	123.3 (6)	O2B—C9B—O3B	123.7 (6)
O2A—C9A—C8A	121.6 (7)	O2B—C9B—C8B	121.4 (7)
O3A—C9A—C8A	115.1 (6)	O3B—C9B—C8B	114.9 (6)
O3A—C10A—H10A	109.5	O3B—C10B—H10D	109.5
O3A—C10A—H10B	109.5	O3B—C10B—H10E	109.5
H10A—C10A—H10B	109.5	H10D—C10B—H10E	109.5
O3A—C10A—H10C	109.5	O3B—C10B—H10F	109.5
H10A—C10A—H10C	109.5	H10D—C10B—H10F	109.5
H10B—C10A—H10C	109.5	H10E—C10B—H10F	109.5
N1A—C11A—C12A	114.9 (7)	N1B—C11B—C12B	116.7 (7)
N1A—C11A—H11A	108.6	N1B—C11B—H11C	108.1
C12A—C11A—H11A	108.6	C12B—C11B—H11C	108.1
N1A—C11A—H11B	108.6	N1B—C11B—H11D	108.1
C12A—C11A—H11B	108.6	C12B—C11B—H11D	108.1
H11A—C11A—H11B	107.5	H11C—C11B—H11D	107.3
C13A—C12A—C11A	125.7 (9)	C13B—C12B—C11B	125.2 (8)
C13A—C12A—H12A	117.2	C13B—C12B—H12B	117.4
C11A—C12A—H12A	117.2	C11B—C12B—H12B	117.4
C12A—C13A—H13A	120.0	C12B—C13B—H13C	120.0
C12A—C13A—H13B	120.0	C12B—C13B—H13D	120.0
H13A—C13A—H13B	120.0	H13C—C13B—H13D	120.0
			
O5A—S1A—N1A—C1A	167.7 (5)	O4B—S1B—N1B—C1B	−64.2 (5)
O4A—S1A—N1A—C1A	−61.2 (5)	O5B—S1B—N1B—C1B	167.3 (5)
C8A—S1A—N1A—C1A	51.4 (5)	C8B—S1B—N1B—C1B	49.4 (5)
O5A—S1A—N1A—C11A	0.2 (6)	O4B—S1B—N1B—C11B	119.2 (5)
O4A—S1A—N1A—C11A	131.4 (5)	O5B—S1B—N1B—C11B	−9.3 (6)
C8A—S1A—N1A—C11A	−116.0 (5)	C8B—S1B—N1B—C11B	−127.1 (5)
C11A—N1A—C1A—C2A	−44.4 (8)	C11B—N1B—C1B—C6B	143.2 (6)
S1A—N1A—C1A—C2A	148.1 (5)	S1B—N1B—C1B—C6B	−33.2 (7)
C11A—N1A—C1A—C6A	132.4 (6)	C11B—N1B—C1B—C2B	−32.6 (9)
S1A—N1A—C1A—C6A	−35.1 (7)	S1B—N1B—C1B—C2B	150.9 (5)
C6A—C1A—C2A—C3A	3.5 (9)	C6B—C1B—C2B—C3B	2.5 (9)
N1A—C1A—C2A—C3A	−179.7 (6)	N1B—C1B—C2B—C3B	178.2 (5)
C1A—C2A—C3A—C4A	−1.6 (10)	C1B—C2B—C3B—C4B	−1.8 (9)
C2A—C3A—C4A—C5A	−1.3 (11)	C2B—C3B—C4B—C5B	0.6 (9)
C3A—C4A—C5A—C6A	2.2 (10)	C3B—C4B—C5B—C6B	−0.2 (9)
C4A—C5A—C6A—C1A	−0.3 (9)	C2B—C1B—C6B—C5B	−2.0 (8)
C4A—C5A—C6A—C7A	−175.5 (6)	N1B—C1B—C6B—C5B	−177.7 (5)
C2A—C1A—C6A—C5A	−2.6 (9)	C2B—C1B—C6B—C7B	173.3 (6)
N1A—C1A—C6A—C5A	−179.4 (5)	N1B—C1B—C6B—C7B	−2.5 (8)
C2A—C1A—C6A—C7A	172.6 (5)	C4B—C5B—C6B—C1B	0.8 (8)
N1A—C1A—C6A—C7A	−4.1 (8)	C4B—C5B—C6B—C7B	−174.5 (6)
C5A—C6A—C7A—O1A	15.4 (8)	C1B—C6B—C7B—O1B	−165.1 (5)
C1A—C6A—C7A—O1A	−159.8 (5)	C5B—C6B—C7B—O1B	9.9 (8)
C5A—C6A—C7A—C8A	−166.3 (6)	C1B—C6B—C7B—C8B	13.5 (9)
C1A—C6A—C7A—C8A	18.6 (9)	C5B—C6B—C7B—C8B	−171.5 (5)
O1A—C7A—C8A—C9A	1.1 (9)	O1B—C7B—C8B—C9B	2.4 (9)
C6A—C7A—C8A—C9A	−177.1 (5)	C6B—C7B—C8B—C9B	−176.1 (5)
O1A—C7A—C8A—S1A	−175.3 (5)	O1B—C7B—C8B—S1B	−170.5 (4)
C6A—C7A—C8A—S1A	6.4 (8)	C6B—C7B—C8B—S1B	11.1 (8)
O5A—S1A—C8A—C7A	−150.2 (5)	O4B—S1B—C8B—C7B	76.5 (5)
O4A—S1A—C8A—C7A	76.4 (5)	O5B—S1B—C8B—C7B	−151.2 (4)
N1A—S1A—C8A—C7A	−37.3 (5)	N1B—S1B—C8B—C7B	−37.6 (5)
O5A—S1A—C8A—C9A	33.4 (6)	O4B—S1B—C8B—C9B	−96.3 (5)
O4A—S1A—C8A—C9A	−100.1 (5)	O5B—S1B—C8B—C9B	36.0 (6)
N1A—S1A—C8A—C9A	146.2 (5)	N1B—S1B—C8B—C9B	149.6 (5)
C10A—O3A—C9A—O2A	4.1 (9)	C10B—O3B—C9B—O2B	3.3 (9)
C10A—O3A—C9A—C8A	−178.7 (5)	C10B—O3B—C9B—C8B	−175.2 (5)
C7A—C8A—C9A—O2A	2.2 (9)	C7B—C8B—C9B—O2B	−0.2 (9)
S1A—C8A—C9A—O2A	178.5 (5)	S1B—C8B—C9B—O2B	172.3 (5)
C7A—C8A—C9A—O3A	−175.0 (5)	C7B—C8B—C9B—O3B	178.3 (5)
S1A—C8A—C9A—O3A	1.2 (8)	S1B—C8B—C9B—O3B	−9.2 (8)
C1A—N1A—C11A—C12A	103.1 (7)	C1B—N1B—C11B—C12B	97.5 (7)
S1A—N1A—C11A—C12A	−89.9 (7)	S1B—N1B—C11B—C12B	−86.1 (7)
N1A—C11A—C12A—C13A	8.8 (11)	N1B—C11B—C12B—C13B	10.2 (11)

### Hydrogen-bond geometry (Å, º)

**Table:**

*D*—H···*A*	*D*—H	H···*A*	*D*···*A*	*D*—H···*A*
O1*A*—H1*OA*···O2*A*	0.82	1.84	2.553 (8)	144
O1*B*—H1*OB*···O2*B*	0.82	1.87	2.588 (8)	146
